# Personalized neoantigen vaccination with synthetic long peptides: recent advances and future perspectives

**DOI:** 10.7150/thno.38742

**Published:** 2020-05-15

**Authors:** Xiaotong Chen, Ju Yang, Lifeng Wang, Baorui Liu

**Affiliations:** The Comprehensive Cancer Centre of Drum Tower Hospital, Medical School of Nanjing University & Clinical Cancer Institute of Nanjing University, Nanjing, China

**Keywords:** Neoantigen, cancer vaccine, long peptide, solid tumor, immunotherapy

## Abstract

Therapeutic cancer vaccines are one of the most promising strategies of immunotherapy. Traditional vaccines consisting of tumor-associated antigens have met with limited success. Recently, neoantigens derived from nonsynonymous mutations in tumor cells have emerged as alternatives that can improve tumor-specificity and reduce on-target off-tumor toxicity. Synthetic peptides are a common platform for neoantigen vaccines. It has been suggested that extending short peptides into long peptides can overcome immune tolerance and induce both CD4^+^ and CD8^+^ T cell responses. This review will introduce the history of long peptide-based neoantigen vaccines, discuss their advantages, summarize current preclinical and clinical developments, and propose future perspectives.

## Introduction

Immunotherapy represents a significant breakthrough in the field of cancer treatment, which aims to harness the immune system to recognize tumor antigens and destroy tumors while leaving normal tissues undamaged 1. Therapeutic cancer vaccines are one of the most promising strategies of immunotherapy 2. In contrast to prophylactic vaccines, therapeutic vaccines are intended to induce robust cell-mediated immunity rather than antibody protection 3. This can be achieved through increasing tumor antigen presentation of the major histocompatibility complex (MHC) expressed on antigen-presenting cells (APCs), allowing a larger number of T lymphocytes to identify and eradicate tumor cells 4. Despite numerous efforts to develop cancer vaccines, their translation into efficacious clinical therapies has been challenging, with less than 7% objective clinical responses and an overall rate of clinical benefit around 20% 5. To achieve the full potential of cancer vaccines, personalized neoantigen vaccines have been introduced 6.

Personalized neoantigen vaccines utilize neoantigens derived from nonsynonymous mutations of tumor cells, which are an important class of tumor antigens mediating anti-tumor immunity in addition to tumor-associated antigens (TAAs) 1. While TAAs are present on tumor cells as well as normal cells, the expression of neoantigens is restricted to tumor tissues 7. As such, improved tumor-specificity and lower levels of on-target off-tumor toxicity can be expected for neoantigen vaccines compared to TAA vaccines 8. In addition, vaccines targeting self-antigens have been shown to elicit T cells with low avidity due to thymic selection and central tolerance 9. However, neoantigens are new to the immune system. High-avidity T cells targeting neoantigens are more likely to exist 10. From this perspective, neoantigen vaccines represent an attractive approach for therapeutic cancer vaccines. Recent clinical trials have demonstrated that T cell responses can be augmented or induced *de novo* by vaccination with predicted neoantigens in melanoma and glioblastoma patients, highlighting their potential as anti-cancer therapeutics 11-15.

Dendritic cell (DC) vaccines, DNA vaccines, RNA vaccines and synthetic peptide vaccines constitute the four platforms of personalized neoantigen vaccines 2. Due to their relatively simple manufacturing process and stable storage 16, synthetic peptide vaccines represent a cost-effective way to generate anti-tumor responses, thereby remaining the therapy of choice for most studies (Table 1). In recent decades, the optimal design of peptide-based vaccines, particularly the size of the vaccinated peptides, has been intensively studied 3. Short peptides typically refer to peptides of 8-10 amino acids in length, which represent the exact minimal CD8^+^ T cell epitopes. They can be extended by natural flanking amino acids to form long peptides, which are generally 15-31 amino acids in length. After vaccination, short peptides directly bind to MHC class I (MHC-I) molecules expressed by all nucleated cells, most of which are not specialized for antigen presentation, thus causing suboptimal T-cell priming or tolerance 17. However, long peptides must be taken up and processed by professional APCs for presentation and T cell activation, thus alleviating potential immune tolerance and enhancing vaccine potency 17, 18. In addition, long peptides involve both CD4^+^ and CD8^+^ T cell responses and have prolonged antigen presentation compared to short peptides 10, 19, 20. Here, we review the history of long peptide-based neoantigen vaccines, elucidate the possible advantages of long peptide vaccines and their mechanisms, summarize their current preclinical and clinical developments, and propose future perspectives.

## History of long peptide-based neoantigen vaccines

**Figure 1:**
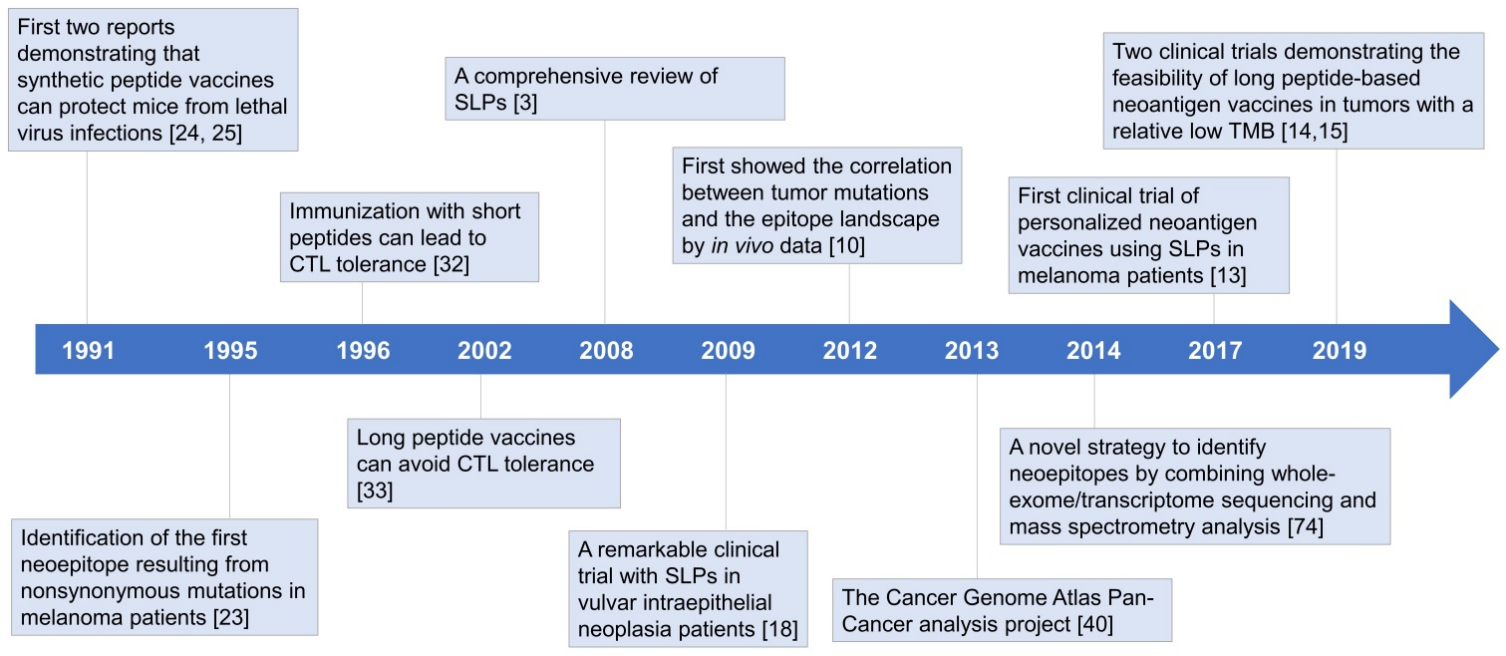
** Historical overview of long peptide-based neoantigen vaccines.** Since the first demonstration that free synthetic peptides could induce protective CTL responses in 1991, considerable efforts have been put into developing peptide-based cancer vaccines, most of which focused on short peptides (8-10 mer) exactly representing the tumor-specific CTL epitopes. However, the clinical translation has met with limited success, and in some cases, peptide vaccination could even accelerate tumor growth. Further exploration revealed that short peptides can lead to immune tolerance, and long peptides (15-31 mer) may act as a more effective platform for therapeutic cancer vaccines. Recent advances in high-throughput sequencing technologies have facilitated the development of personalized vaccines targeting neoantigens derived from nonsynonymous mutations in tumor cells, where long peptides are extensively used. In 2017, the first clinical trial of long peptide-based neoantigen vaccines reported encouraging outcomes in melanoma patients. Subsequent clinical trials have indicated the feasibility in immunologically cold tumors with a relatively low TMB. Emerging data has suggested that neoantigen vaccination with long peptides is a promising strategy to induce potent anti-tumor immunity. CLT: cytotoxic T lymphocyte; SLPs: synthetic long peptides; TMB: tumor mutation burden.

CD8^+^ T lymphocytes have long been regarded as the predominant effector cells in tumor-rejection activities 21. Since the binding grooves of MHC-I molecules are closed at both ends, CD8^+^ T cell epitopes binding to MHC-I molecules are typically restricted to 8-10 amino acids in length. In MHC class II (MHC-II) molecules, the binding grooves are open, which allows the peptides to extend out of the binding grooves, resulting in length diversity (13-25 amino acids) and binding promiscuity of MHC-II ligands. It is much more complicated to predict MHC-II epitopes 22. As a result, researchers initially focused on short peptide vaccines targeting CD8^+^ T cells 23-25. The first two experiments demonstrating the protective effects of peptide vaccines emerged in 1991 24, 25. Immunizing mice with free synthetic peptides could not only generate cytotoxic T lymphocyte (CTL) responses but also induce protection against lethal virus infections. However, it was soon appreciated that not all peptide vaccines could induce strong T cell responses 26-28. In some cases, tumor growth was accelerated after vaccination 29. The outcomes of clinical trials were also disappointing, highlighting the need for alternative strategies to improve therapeutic efficiency (Figure 1) 5, 30.

In the early 21st century, a team from Leiden University Medical Center made a conceptual breakthrough that the size of peptides matters 3. They pointed out that some successful studies did not necessarily use the exact short peptides of 8-10 amino acids in length but may be longer 24, 31. Subsequent experiments elucidated that short peptide vaccines could induce CTL tolerance (discussed below) 17, 19, 29, 32, 33, explaining the inconsistency between studies. Great success was achieved in 2009 in vulvar intraepithelial neoplasia patients, further highlighting the potential of synthetic long peptides (SLPs) as a vaccine platform (Figure 1) 18. In this clinical trial, 15 of 19 patients showed clinical responses, with complete responses in 9 of them. These complete responses were maintained at 24 months of follow-up 18.

Likewise, “neoantigen” is not a new concept. Since the identification of the *src* oncogene in the 1970s 34, scientists gradually realized that cancer is a genetic disease, and that malignant transformation was caused by mutations in proto-oncogenes or tumor suppressor genes, resulting in their abnormal expression. The protein products of these mutated genes became candidate cancer neoantigens 35. It was first shown in 1995 that neoantigens purified from murine lung carcinoma could be converted into peptide vaccines that were therapeutically effective, enhancing the lifespan of mice by protecting them from metastasis 23, 36. Similar conclusions were achieved in melanoma patients 37. However, traditional neoantigen vaccines typically consisted of a single mutant peptide corresponding to only one hotspot mutation (e.g. *KRAS* codon 12 mutations) 38. Despite their improved tumor-specificity and lower on-target off-tumor toxicity, the clinical translation of such vaccines remains in its infancy 39.

Rapid developments in high-throughput sequencing and bioinformatics have facilitated the comprehensive mapping of all mutations in a tumor, termed the “mutanome” 40. In 2012, Matsushita et al. validated the feasibility of combining next-generation sequencing (NGS) and predictive algorithms to identify MHC class I-restricted neoepitopes in a mouse sarcoma model 41. Soon the mode of manufacturing personalized neoantigen vaccines was established, which incorporated exome and RNA sequencing of tumor and normal tissues to identify somatic mutations, followed by computational prediction and prioritization of neoepitopes (Figure 1) 10. Generally, neoepitopes are predicted according to their HLA binding affinity 42. Other factors accounting for immunogenicity are also considered in neoantigen prediction and prioritization, including proteasome cleavage preference 43, gene expression and peptide abundance 44, and structural and physicochemical features of peptide-MHC complexes 45. Neoantigen vaccines derived from this strategy differ from the traditional version in that they consist of several top ranked epitopes rather than a single mutant peptide to deal with tumor heterogeneity and avoid immune escape 13-15. Moreover, they are truly personalized due to the diversity of each individual's mutanome. The multi-epitope neoantigen vaccines are customized for each individual patient. Recent studies have shown attractive prospects, where SLPs are extensively used 10, 13, 14. SLPs vary from 15 to 31 amino acids in length, composed of a predicted MHC-I epitope elongated at both ends with natural residues 10, 13-15. This design enables all potential MHC-I or MHC-II epitopes of 8-15 amino acids in length carrying the mutation to be processed from the precursor peptide 10. Peptides binding to the same MHC molecules are separated into different pools, administered in a non-rotating fashion to one of up to four extremities, avoiding potential antigen competition in the draining lymph nodes (dLNs) 13, 14, 46.

## Advantages of long peptide-based neoantigen vaccines

### Overcoming potential CTL tolerance

The substantial differences between long and short peptide vaccines originate from their presentation to APCs. The most distinctive feature of minimal CTL epitope vaccines is their direct binding to MHC molecules. Theoretically, minimal CTL epitopes are expected to be loaded directly onto MHC-I molecules expressed by local submucosal DCs at the injection site 47. These DCs migrate to the dLNs where they present antigens to naive T cells, stimulating them to differentiate into antigen-specific CD8^+^ effector T lymphocytes 17. Chemokines and other signaling factors further recruit these CTLs to tumor sites 48. Thus, tumor-specific CTL responses are generated and anti-tumor responses are initiated.

**Figure 2:**
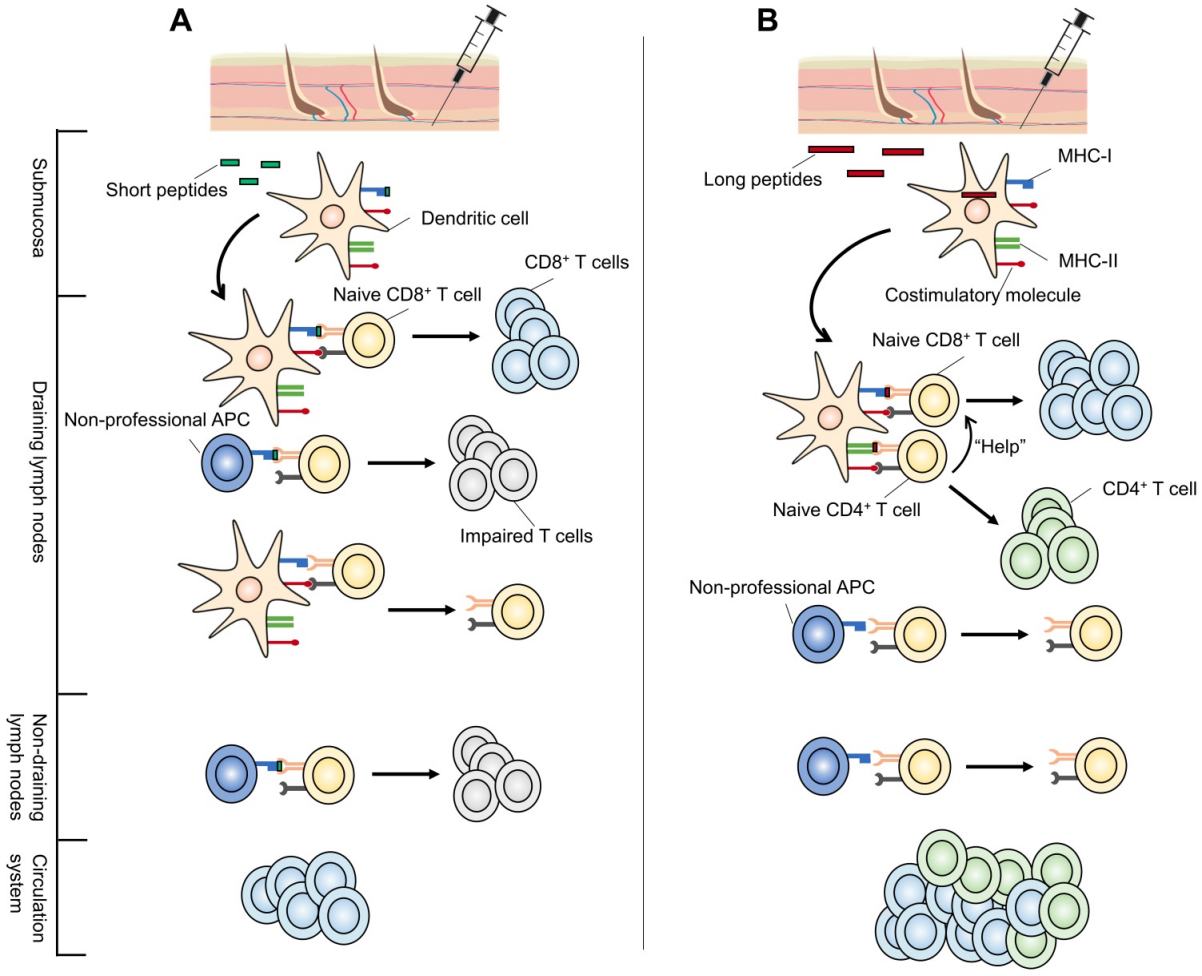
** Possible mechanisms for the superior performance of long peptide-based neoantigen vaccines *vs* short peptides.** (**A**) Short peptide neoantigen vaccines (green) bind to MHC class I molecules expressed on local submucosal DCs once injected. These DCs migrate to the dLNs to present and activate naive T cells. However, short peptides with low MHC-binding affinity may fail to elicit a robust CTL response. In addition, short peptides can be presented systemically in not only dLNs but also ndLNs by all nucleated cells, most of which are not specialized for antigen presentation. Lack of costimulatory molecules on those non-professional APCs and improper stimulating environments (ndLNs) can both result in impaired T cell function. (**B**) Long peptides (red) must be endocytosed and processed for their transport to the cell surface in a DC-focused pattern. They are presented predominantly in dLNs. In addition, long peptide neoantigen vaccines may cover CD4^+^ T cell epitopes, involving CD4^+^ T_h_ responses which play an important role in neoantigen anti-tumor immunity. Subsequently, they exhibit superior performance over short peptide neoantigen vaccines. APCs: antigen-presenting cells; CTL: cytotoxic T lymphocyte; DC: dendritic cell; dLNs: draining lymph nodes; MHC: major histocompatibility complex; ndLNs: non-draining lymph nodes.

However, the ability of a short peptide to be presented in dLNs depends on its ability to remain bound to MHC molecules. Short peptides displaying low MHC-binding affinity often show difficulties in eliciting a robust CTL response (Figure 2). Furthermore, peptides can diffuse from the vaccine site and spread systemically, and MHC-I molecules are expressed on the surface of all nucleated cells 49. Consequently, short peptides can be directly loaded onto the MHC-I molecules of various types of professional APCs and non-professional APCs, most of which lack a full range of costimulatory molecules required for optimal CD8^+^ T cell activation 19. As a result, short peptides often activate CTLs transiently or even induce CTL tolerance 17, 19, 33, 50. For example, short peptide vaccination was shown to result in antigen presentation by circulating lymphocytes (including B cells and T cells) in not only dLNs but also non-draining lymph nodes (ndLNs) in the absence of a strong pro-inflammatory context (Figure 2A) 19, 51. Even when activated by CpG oligodeoxynucleotides (CpG-ODN), they still failed to generate therapeutically efficient anti-tumor immunity 19.

Elongating short peptides with natural flanking amino acids into SLPs alters this procedure (Figure 2B). SLPs elicit stronger effector CTL responses with greater tumoricidal potential in a DC-focused pattern 51-53, which is regarded as the main cross-presentation APC *in vivo* 54. SLPs must be endocytosed, processed by DCs, and transported to cell surface rather than directly binding to MHC molecules 51. Following internalization, a proportion of SLPs are degraded through the endosomal pathway and loaded onto MHC-II molecules, permitting their recognition by CD4^+^ T helper cells (T_h_ cells) 47. Another part of endocytosed SLPs enter either the cytosol or vacuolar pathway and are cross-presented by MHC-I molecules, activating CD8^+^ CTLs 54, 55. This processing-dependency of SLPs to generate antigen-specific CD8^+^ T cell responses circumvents the possible CTL tolerance mechanisms 51. Besides, data showed that, in contrast to systemically presented short peptides, long peptides are presented predominantly in dLNs 19. These findings make it reasonable to believe that a stronger and more effective response can be induced with long peptide vaccines. In fact, the efficacy of this superior tumor-specific immunity has been demonstrated in a preclinical model of human papillomavirus (HPV) 16-induced cervical cancer. The eradication of large, established HPV16-expressing tumors was accomplished using a 35-mer long peptide admixed with the DC-activating adjuvant CpG-ODN, but not with a 9-mer short peptide containing the same CTL epitope 33.

### Involving CD4^+^ T_h_ cell responses

CD8^+^ cytotoxic T cells (T_c_ cells) have been intensely studied in anti-tumor immunity 21. However, over recent decades, emerging evidence has shown that a deficiency of CD4^+^ T_h_ cells impairs CTL responses, indicating their indispensable role 56-58. CD4^+^ T_h_ cells can license DCs through CD40-CD40L interaction, generating more efficient antigen presentation to CTLs 59. Despite their traditional role in the immune response through cytokine secretion 60, CD4^+^ T cells exhibit cytotoxic features and can directly eliminate tumors in the absence of an MHC-I restricted CD8^+^ T cell response 57, 58, 61 . Surprisingly, when it comes to personalized neoantigen vaccines, it has been suggested that the majority of the immunogenic mutanome is recognized by CD4^+^ T cells in tumor-bearing C57BL/6 mice 62, further emphasizing the necessity of involving CD4^+^ T cell responses in anti-tumor immunity. Meanwhile, prominent CD4^+^ T cell responses were also shown in recently published neoantigen vaccine clinical trials, despite the use of MHC-I binding prediction algorithms 12-14.

It seems that short peptides can activate CD8^+^ T cells more easily than SLPs since short peptides can be directly presented on MHC-I molecules after vaccination, skipping the endocytosis and intracellular processing steps 51, 63. However, this superior antigen presentation efficacy of short peptides to that of SLPs can only be observed early after vaccination, and this phenomenon is reversed with time due to the long-lasting cross-presentation of SLPs (discussed below) 63. Furthermore, SLPs allow the generation of various combinations of the T_h_ or T_c_ epitopes containing the mutated amino acid^10^. While vaccination with mixed T_c_ and T_h_ epitopes can achieve the same goal 64, 65, chemically linked T_h_ and T_c_ epitopes further increased the magnitude of the CTL response, suggesting that it is more efficient to provoke anti-tumor immunity by presenting the two epitopes on the same APC rather than on different APCs 59, 66, 67. But this method still risks failure as alterations in the amino acid terminus to link the two epitopes together may lead to inappropriate cleavage by the proteasome, directly affecting epitope presentation 68. In addition, algorithms to predict MHC-II restricted neoepitopes are still in their infancy 69. Therefore, there is a tendency to vaccinate with SLPs to provide a potential class II epitope(s) so as to involve both CD4^+^ and CD8^+^ T cell responses 59, 70.

### Prolonging antigen cross-presentation

Short peptides directly bind to MHC-I molecules expressed on local DCs once injected, forming MHC class I-peptide complexes to prime antigen-specific CD8^+^ T cells 47. However, MHC class I-peptide complexes have a high turnover at the cell surface of mature DCs. Most MHC class I-peptide complexes can hardly be detected on the cell surface of mature DCs after 24 h, while MHC class II-peptide complexes are stable for several days 20. This is because that the function of MHC-I molecules is to continuously present ligands derived from cytosolic proteins, newly synthesized mis-folded proteins and/or viral proteins for the timely elimination of abnormal tissue cells and pathogens 71. As a result, the duration of antigen presentation of short peptide vaccines is limited, which is considered to partly account for their suboptimal efficiency 19.

However, antigen cross-presentation by MHC-I molecules of SLPs remained detectable for at least 3 days, correlating with an increased magnitude of anti-tumor responses 19, 20. Further investigations showed that antigens which need internalization and intracellular processing to release MHC-I ligands (such as SLPs) can be conserved in intracellular storage depots of DCs for several days. This ensures a continuous supply of antigens and contributes to their sustained cross-presentation by DCs, despite the high turnover of MHC class I-peptide complexes at the cell surface 20. Storage organelles were characterized as lysosome-like compartments 20. Additional experiments are required for more detailed descriptions regarding these antigen depots. Taken together, SLPs which can be persistently cross-presented to activate CTLs may be superior to short peptides that are rapidly lost from MHC-I molecules in vaccine formulations.

## Current studies of long peptide-based neoantigen vaccines

### Preclinical studies

Bijker et al. performed a comparison of different peptide vaccination strategies with the highly immunogenic model antigen OVA, and showed a superior performance of long peptides over short peptides 17. The minimal T_c_ epitope OVA_257-264_ (OVA8), the T_h_ epitope OVA_323-339_ (OVA17), the extended T_c_ epitope OVA_241-270_ (OVA30) and the extended T_h_ epitope OVA_317-347_ (OVA31) were prepared. Injecting OVA8 alone in incomplete Freund's adjuvant (IFA) induced a transient CD8^+^ response but failed to undergo a secondary expansion 30 days later, while OVA17 alone was sufficient to induce both short-term (10 days) and long-term (30 days) responses. The addition of the T_h_ epitope OVA17 to OVA8 retained CD8^+^ T cell functionality, indicating an important role of CD4^+^ T cells in generating memory T cells. Long-lasting CD8^+^ T cell immunity was also observed when the extended T_c_ epitope OVA30 was injected alone. Depletion of CD4^+^ T cells did not influence the cytotoxic capacity of OVA30-induced CTLs. The number of OVA-specific CD8^+^ CTLs after expansion was higher when combined with OVA31 or an agonistic CD40 antibody. The greater capability of long peptides to induce anti-tumor immunity was related but not limited to CD4^+^ T cell responses. Similar results were confirmed in other preclinical models 19, 33, 72.

Based on these observations, Castle et al. made the first attempt to manufacture personalized neoantigen vaccines employing the long peptide platform in a high-throughput way 10. DNA and RNA of matched tumor and normal tissues were extracted from B16F10 mice. Whole-exome sequencing (WES) was performed and their expression were further validated via RNA sequencing (RNA-Seq). In total, 563 mutations in expressed genes were identified, 50 of which were selected for synthesis into peptides of 27 amino acids long with the mutated or wild-type amino acid on position 14. By vaccinating C57BL/6 mice subcutaneously with polyinosinic-polycytidylic acid stabilized with polylysine and carboxymethylcellulose (poly-ICLC) as adjuvants, 16 out of 50 mutations were immunogenic and 60% in this group evoked strong immune responses directed preferentially against the mutated sequence rather than the wild-type sequence. These neoantigen vaccines also confirmed *in vivo* tumor control in protective and therapeutic settings. Furthermore, compared to vaccines comprised of the minimal 8-mer epitopes, long peptide-based personalized neoantigen vaccines showed improved protection during a tumor rechallenge analysis 73.

To simplify the laborious screening of immunogenic mutant epitopes, Yadav et al. established a new strategy to identify neoepitopes 74. After WES and RNA-seq, mass spectrometry (MS) analysis was used to recognize the truly presented peptides by MHC-I molecules in MC-38 and TRAMP-C1 mice tumor cell lines, followed by a structural prediction algorithm to predict MHC-I peptide immunogenicity. Compared to the 170 and 6 neoepitopes respectively suggested in 2 tumor cell lines by direct algorithm prediction after RNA-seq, 5 and 0 neoepitopes were predicted using this strategy. Of these, 3 were confirmed as immunogenic through T cell analysis. The authors also employed the long peptide platform and successfully validated the feasibility and effectiveness of the neoantigen vaccines developed by this method 74.

Other than the highly personalized neoantigens identified through the two strategies described above, some neoantigens are encoded by recurrent driver mutations and hence are shared between patients 75. Through literature reviews and database analysis, one can also identify such shared neoantigens to develop corresponding vaccines. Schumacher et al. selected the mutation IDH1(R132H) that is expressed in more than 70% diffuse grade II and III glioma patients as a target for neoantigen vaccines 61. They generated peptide libraries encompassing this mutation and demonstrated that, the long peptide p123-142 (R132H) vaccine exhibited an anti-tumor immunity that was equivalent to another peptide vaccine targeting a well-studied tumor-associated antigen: NY-ESO-1. This study also represented the strong CD4^+^ immune responses which can reject tumors independent of CD8^+^ T cell responses 61, 76, 77. The three approaches mentioned above represent the major screening methods to develop neoantigen vaccines, all of which have been validated through long peptide vaccines, demonstrating their recognition, immunogenicity, tumor-specificity and *in vivo* protective effects.

### Clinical studies

Currently, five clinical trials of personalized neoantigen vaccines have been published (Table 1), three of which employed long peptides 13-15. The other two were DC 11 and RNA vaccines 12. Sahin et al. engineered two synthetic RNAs, each encoding five linker-connected 27-mer peptides with mutations at position 14 12. These RNA vaccines were eventually translated into long peptides, and to some extent, were consistent with the idea of long peptide vaccines, wherein the translated peptides are loaded on to the MHC intracellularly, and then exported to the cell surface for presentation to T cells 78.

Ott et al. prepared up to 20 long peptides for each melanoma patient that were 15-30 amino acids in length and divided into 4 pools 13. Of the six vaccinated patients, four remained recurrence-free at 25 months post-vaccination, while two with progresssive disease were subsequently treated with anti-PD-1 therapy and experienced complete tumor regression. These results were astonishing and provided a strong rationale for further exploration to combine immune checkpoint inhibitors with neoantigen vaccines 13. Keskin et al. from the same team of Dana-Farber Cancer Institute adopted a similar vaccination scheme in methylguanine methyltransferase (MGMT)-unmethylated glioblastoma patients 14. Their results showed that long peptide-based neoantigen vaccines were a feasible therapeutic strategy for immunologically cold tumors with a relatively low tumor mutation burden (TMB) 14. However, all patients died of progressive disease with a median progression-free survival of 7.6 months and overall survival of 16.8 months. Hilf et al. investigated glioblastoma therapeutics with a different design 15. They used short peptides targeting unmutated TAAs derived from a premanufactured library, followed by the 19-mer neoepitope vaccination. The median progression-free survival was 14.2 months and the overall survival was 29.0 months 15. This study investigated the clinical outcome of short TAA-targeting epitopes incorporating long neoantigen-targeting epitopes with encouraging results. However, all these trials revealed that considerable challenges still remain and further exploration is required to achieve the optimal design of neoantigen vaccines for ideal therapeutic effects.

## Future perspectives

Despite the remarkable anti-tumor potential of long peptide-based neoantigen vaccines shown in both preclinical and clinical settings, the total results remain far from satisfying. Generally, there are four critical issues during vaccine design: (1) antigen selection; (2) adjuvant utilization; (3) vaccine delivery methods; (4) immune suppression reversion. We herein propose some improving approaches according to these four aspects.

### Improving antigen prediction

The common workflow to create a personalized neoantigen vaccine includes exome and transcriptome sequencing of matched tumor-normal tissues, followed by *in silico* prediction and prioritization of neoepitopes 79. Great progress has been made in the methodologies employed by neoantigen predictors, shifting from scoring function-based tools to machine learning-based tools 80, but there is still significant room for improvement.

Firstly, neoantigens can be generated from various sources beyond single nucleotide variants (SNVs), including frameshift mutations 81, gene fusions 82, intron retentions 83, non-coding expressed regions 84 and post-translational modifications 85. However, most predictors only identify neoantigens from SNVs, leaving many highly immunogenic neoepitopes undiscovered 69. Secondly, MHC-II alleles are inadequately supported in current prediction tools due to the variable length and binding promiscuity of MHC-II ligands and lack of binding data for model training 86. However, it has been recently appreciated that neoantigen-specific responses are mediated predominantly by CD4^+^ T cells, highlighting the essential role of accurate MHC-II predictions 12-14. In addition, a majority of predictors predict candidate neoepitopes according to their binding affinity of MHC molecules 69, without the consideration of other factors contributing to the immunogenicity such as proteasomal cleavage and peptide transportation 43, stability and T-cell receptor (TCR) recognition of the peptide-MHC complexes 87, and structural and physicochemical features 45, etc.

In recent years, while significant technological improvements have extended neoantigen identification to indels (tools like Strelka 88, EBCall 89) and gene fusions (tools like JAFFA 90, INTEGRATE 91), other newly-emerged sources remain to be involved. Specialized bioinformatics tools have also been developed to predict MHC-II antigen presentation. Two recently published algorithms (MARIA 86 and MixMHC2pred 92) were reported to outperform existing methods, including NetMHCIIpan and SMM Align, which are commonly used in MHC-II restricted neoepitope prediction at present 69. Moreover, MHC-I and MHC-II algorithms can be combined for more accurate predictions 44. pVACtools is a comprehensive and extensible toolkit that can identify neoantigens from SNVs, indels and gene fusions. It integrates eight MHC-I and four MHC-II algorithms, supporting stability and cleavage predictions. pVACtools can be used for the design of long peptide-based vaccines, assessing candidate SLPs by evaluating their manufacturability (NCT03122106) 44. Other specialized algorithms include NetChop for peptide processing prediction 43, DeepHLApan for TCR recognition prediction 87, and TRUST for TCR repertoire profiling 93, all of which have been well summarized in other reviews 94, 95. With these fast updating bioinformatics tools, personalized neoantigen vaccines will be more accessible to patients, especially those with low TMB.

### Engaging novel adjuvants

Since their first description by Ramon in 1924, diverse classes of adjuvants have been developed. Examples in current stages of development are listed in Table 3 (data from clinicaltrials.gov). Recent studies have reported that traditional adjuvants such as IFA and aluminum salts may induce T cell retention, exhaustion and deletion based on observations using short peptide vaccines 96. Although this may not be the case for long peptides 3, more powerful adjuvants have been explored.

Toll-like receptor (TLR) agonists have been extensively investigated (Table 3). Poly-ICLC acts as a TLR3 agonist and has shown promising prospects. In 3 out of 5 published neoantigen clinical trials used poly-ICLC, strong T cell responses were demonstrated 13-15. Among 36 ongoing clinical trials of peptide neoantigen vaccines, 20 selected poly-ICLC as adjuvants (Table 2). Agonist antibodies targeting CD40 expressed on DCs represent another attractive approach to improve the activation of DCs and induce superior immune responses. APX005M is among the six CD40 agonists currently under development (Table 3). It is in a phase I study of melanoma patients, combined with neoantigen peptide vaccines (NEO-PV-01) and immune checkpoint inhibitors (NCT03597282). Stimulator of interferon genes (STING) is an endoplasmic reticulum adaptor first described in 2008 97. Subsequent elucidation of downstream signaling pathways highlighted its potential as a target for cancer immunotherapy that can activate innate immunity 98. Clinical studies incorporating STING agonists and peptide vaccines are still lacking , but their potent ability to function as an adjuvant with a whole-cell tumor cell vaccine have been demonstrated in mice 99.

### Employing nanodelivery systems

Nanoparticles (NPs) for drug delivery have long been an attractive therapeutic strategy 100. By employing the nanovaccine delivery system, we can: (1) protect peptides from rapid degradation to prolong their presentation time; (2) increase the accumulation of peptides in lymphatic tissue and improve the co-delivery of antigen peptides and adjuvants to dLNs; (3) deliver antigens and adjuvants simultaneously to DCs and promote their internalization 101. Intracellular delivery is particularly important for long peptide-based vaccines as they must undergo endocytosis 3. Moreover, some pattern recognition receptors (PRRs) are expressed inside the cell, such as TLR3, TLR7, TLR8, TLR9 and STING 102. Delivering vaccines in the form of NPs can therefore improve immune activation and achieve optimal results.

Kuai et al. designed a synthetic high-density lipoprotein (sHDL) nanodisc, of which the surface was decorated with neoantigen long peptides and the TLR9 agonist CpG motif. This nanodisc generated 47-fold greater frequencies of neoantigen-specific CTLs than soluble vaccines with CpG as an adjuvant. Moreover, established MC-38 and B16F10 tumors were eliminated when combined with anti-PD-1 and anti-CTLA-4 therapies 103. Recently, Li et al. reported a simple adsorption strategy using polyethyleneimine (PEI) in a mesoporous silica micro-rod (MSR) vaccine approach to enhance antigen response for neoantigen vaccines, with granulocyte-macrophage colony-stimulating factor (GM-CSF) and CpG-ODN as adjuvants. A single injection of this vaccine using a synthetic long peptide derived from the HPV E7 oncoprotein completely eradicated large established TC-1 tumors in ~80% of mice and generated immunological memory 104. Furthermore, special biomaterials or novel designs are now under rapid development, which allow nanovaccines to respond to certain environmental triggers such as pH, redox, light or ultrasound. Improved DC-targeting, cytosolic delivery and therapeutic efficiency have been demonstrated 105. Wang et al. designed a carrier-free nanovaccine with a high antigen density. Intermolecular disulfide cross-linking between antigens formed a nanoscale network. CpG bearing a thiol group was further incorporated into this network as a “danger signal” to activate DCs. Upon taken up by DCs, intracellular enriched glutathione (GSH) mediated the cleavage of the disulfide bonds, resulting in the release of antigens and CpG. This nanovaccine significantly promoted antigen-specific T cell activation with enhanced dLN retention, showing a higher survival rate of C57BL/6 mice and successful induction of tumor prevention 106.

### Combining with other therapies

While great efforts have been made in perfecting the design of neoantigen vaccines, immune escape remains a problem that restricts clinical efficacy 107. Tumors may evolve through a set of complex resistance mechanisms under the strong selection pressure of neoantigen-targeting immunotherapies 108, leading to the need for a combination of different therapeutic strategies.

One challenge is the loss or decreased expression of the recognized neoantigen in tumor cells 109. This may be addressed through delivering vaccines consisting of multiple neoepitopes to induce polyclonal immune responses, as performed in many studies of neoantigen vaccines 13-15. DNA-damaging chemoradiotherapies can act as powerful mutagens to introduce new somatic mutations and convert the tumor into an *in situ* vaccine, adding to the efficiency of neoantigen vaccines 110, 111. Furthermore, vaccines targeting TAAs may also serve as a complement especially in patients with low TMB, as shown by Hilf et al 15.

The downregulation of components of antigen presentation machinery such as MHC-I molecules and the transporter associated with antigen processing (TAP) is the most frequently observed immune evasion mechanism that results in impaired antigen presentation 112.

Therapeutic kinase inhibitors targeting MEK and EGFR may have synergistic effects with neoantigen vaccines since they can upregulate MHC-I and TAP expression and enhance antigen presentation 113. Epigenetic modulators such as DNA methyltransferase inhibitors can be considered for combination as well according to the epigenetic repression mechanisms of MHC expression 107. Moreover, tumor cells lacking antigen presentation can be additionally eliminated in an MHC-independent fashion either by adoptive transfer of chimeric antigen receptor T (CAR-T) cells 114 or through the induction of antibody-mediated activation of natural killer cells 115.

Immune-inhibitory tumor microenvironment (TME) is another important factor hampering the performance of neoantigen vaccines 116. Although lymphocytes can be efficiently activated by long peptides in the peripheral blood, reduced adhesion molecules due to abnormal angiogenesis and increased extracellular matrix density in tumor tissues prevent effective T cell migration and infiltration. Local immunosuppressive cells and molecules also compromise neoantigen recognition and T cell activation 117. Combination strategies incorporating anti-angiogenesis therapies can normalize tumor vessels and reprogram suppressive TME, promoting T cell infiltration 118. Immunomodulatory antibodies, including immune checkpoint inhibitors and costimulatory molecule agonists, hold great promise to reverse immune suppression and are under rapid development (Table 4). Complete remission has been achieved in progressed melanoma patients by combining PD-1 inhibitors (described above) 12, 13. In addition, some chemotherapeutic agents (e.g., cyclophosphamide) that deplete immunosuppressive cells are actively being investigated as complementary therapies (NCT03219450, NCT03380871, NCT03606967) 119.

## Conclusion

Personalized neoantigen vaccines show improved tumor specificity and immunogenicity compared to conventional TAA vaccines. Long peptides are widely employed in neoantigen vaccines as a substitute for short peptide-based vaccines to overcome potential immunological tolerance, elicit not only CD8^+^ T cell responses but also CD4^+^ lymphocyte responses and prolong the antigen cross-presentation. Although preclinical experiments and clinical trials of long peptide-based neoantigen vaccines have indicated promising results, additional efforts are warranted to meet the expectations of therapeutic cancer vaccines. Improvements can be made through optimizing antigen prediction, engaging novel adjuvants, employing advanced nanodelivery systems and combining with immunomodulatory antibodies and/or traditional therapies. In summary, a new era of long peptide-based neoantigen vaccines has come and the results of ongoing clinical trials are eagerly anticipated.

## Figures and Tables

**Table 1:** Published clinical trials of personalized neoantigen vaccines

Year	Cancer type	Phase	Formulation	Additional intervention	Vaccine platform	Patient number	Response
2015 11	Melanoma	I	/	/	DC vaccine	3	1 CR 2 SD
2017 12	Melanoma	I	/	/	RNA vaccine	13	8 recurrence free 12-23m; 5 relapse:2 CR with pembrolizumab, 1 PR, 1 mixed response, 1 SD
2017 13	Melanoma	I	Poly-ICLC	/	Long peptide vaccine	6	4 recurrence free 20-32m; 2 relapse: CR with pembrolizumab
2019 14	Glioblastoma	I/Ib	Poly-ICLC	/	Long peptide vaccine	8	8 PD, died; PFS 7.6m, OS 16.8m;
2019 15	Glioblastoma	I	Poly-ICLC GM-CSF	Chemotherapy	Long peptide and short peptide vaccine	15	8 PD, died; PFS 14.2m, OS 29.0m;

CR: complete response; DC: dendritic cell; GM-CSF: granulocyte-macrophage colony-stimulating factor; OS: overall survival; PD: progressive disease; PFS: progression-free survival; Poly-ICLC: polyinosinic-polycytidylic acid stabilized with polylysine and carboxymethylcellulose; PR: partial response; SD: stable disease.

**Table 2:** Ongoing clinical trials of peptide-based neoantigen vaccines (data from ClinicalTrials.gov)

ClinicalTrials.gov Identifier	Cancer type	Phase	Recruitment status	Formulation	Additional intervention
NCT03662815	Advanced Malignant Solid Tumor	I	Recruiting	GM-CSF	/
NCT03645148	Pancreatic Cancer	I	Recruiting	GM-CSF	/
NCT03558945	Pancreatic Tumor	I	Recruiting	Poly-ICLC	/
NCT03715985	Melanoma/NSCLC /Kidney Cancer	I	Recruiting	CAF09b	Anti-PD-1/anti-PD-L1
NCT01970358	Melanoma	I	Active, not recruiting	Poly-ICLC	/
NCT03422094	Glioblastoma	I	Recruiting	Poly-ICLC	Nivolumab/ipilimumab
NCT03068832	Pediatric Brain Tumor	I	Not yet recruiting	Poly-ICLC	/
NCT03361852	Follicular Lymphoma	I	Not yet recruiting	Poly-ICLC	Rituximab
NCT02287428	Glioblastoma	I	Active, not recruiting	/	Radiation/pembrolizumab/temozolomide
NCT02950766	Kidney Cancer	I	Not yet recruiting	Poly-ICLC	Ipilimumab
NCT03606967	TNBC	II	Not yet recruiting	Poly-ICLC	Durvalumab/nab-paclitaxel
NCT03219450	chronic lymphocytic leukemia	I	Not yet recruiting	Poly-ICLC	Cyclophosphamide
NCT03359239	Urothelial/Bladder Cancer	I	Recruiting	Poly-ICLC	Atezolizumab
NCT03559413	Acute lymphoblastic leukemia	I/II	Recruiting	GM-CSF/Imiquimod	
NCT03380871	NSCLC	I	Recruiting	Poly-ICLC	Pembrolizumab/carboplatin/pemetrexed
NCT03597282	Melanoma	I	Recruiting	Poly-ICLC	Ipilimumab/nivolumab/APX005M
NCT02897765	Urinary Bladder Cancer/NSCLC/Melanoma	I	Active, not recruiting	Poly-ICLC	Nivolumab
NCT02992977	Advanced Cancer	I	Active, not recruiting	QS-21 Stimulon®	/
NCT03673020	Solid Tumor, Adult	I	Recruiting	QS-21 Stimulon®	/
NCT03633110	Melanoma/NSCLC/HNSCC /Urothelial Carcinoma/Renal Cell Carcinoma	I/II	Recruiting	Poly-ICLC	Nivolumab
NCT03631043	Smoldering Plasma Cell Myeloma	I	Recruiting	/	/
NCT02600949	Pancreatic /Colorectal Cancer	I	Active, not recruiting	/	Pembrolizumab
NCT02721043	Solid Tumors	I	Recruiting	Poly-ICLC	Lenalidomide
NCT02933073	Ovarian Cancer	I	Recruiting	/	/
NCT03929029	Melanoma	I	Not yet recruiting	Montanide	Ipilimumab/ Nivolumab
NCT04087252	Cancer	I	Recruiting	/	/
NCT03956056	Pancreatic Cancer	I	Not yet recruiting	Poly-ICLC	/
NCT04117087	Pancreatic /Colorectal Cancer	I	Not yet recruiting	Poly-ICLC	Ipilimumab/ Nivolumab
NCT04072900	Melanoma	I	Not yet recruiting	rhGM-CSF	Toripalimab/ Imiquimod
NCT03953235	NSCLC/ Pancreatic /Colorectal Cancer	I/II	Recruiting	/	Ipilimumab/ Nivolumab
NCT03639714	NSCLC/ Colorectal Cancer /Gastroesophageal Adenocarcinoma/Urothelial Carcinoma	I/II	Recruiting	/	Ipilimumab/ Nivolumab
NCT04024878	Ovarian Cancer	I	Not yet recruiting	Poly-ICLC	Nivolumab
NCT03568058	Advanced Cancer	I	Recruiting	/	Pembrolizumab
NCT03121677	Follicular Lymphoma	I	Recruiting	Poly-ICLC	Rituximab
NCT04266730	NSCLC/HNSCC	I	Not yet recruiting	Poly-ICLC	/
NCT04248569	Fibrolamellar Hepatocellular Carcinoma	I	Not yet recruiting	Poly-ICLC	Ipilimumab/ Nivolumab

GM-CSF: granulocyte-macrophage colony-stimulating factor; HNSCC: head and neck squamous cell carcinoma; NSCLC: non-small cell lung cancer; Poly-ICLC: polyinosinic-polycytidylic acid stabilized with polylysine and carboxymethylcellulose; TNBC: triple-negative breast carcinoma.

**Table 3:** Common cancer vaccine adjuvants and their development stages

Classification	Examples under investigation		Stage of development
**Emulsions**	Incomplete Freund's Adjuvant	Montanide ISA51	Phase III
		Montanide ISA720	Phase I
**Mineral salts**	Aluminum salts	Aluminum hydroxide (Alhydrogel™)	FDA approved
		Aluminum phosphate (Adjut-phos™)	FDA approved
**Cytokines**	IL-2	Aldesleukin	FDA approved
	GM-CSF	Sargramostim	FDA approved
	IFNs	Intron A	FDA approved
		Sylatron	FDA approved
**Saponin-based adjuvants**		QS-21	Phase III
		ISCOMATRIX	Phase II
**TLR agonists**	TLR2 agonist	Pam_3_CSK4	Preclinical
	TLR3 agonist	Poly-ICLC	Phase II
	TLR4 agonist	MPLA	Phase II
	TLR7/8 agonist	Imiquimod	FDA approved
		Resiquimod	Phase II
	TLR9 agonist	CpG-ODN	Phase II
**DC-targeted monoclonal antibodies**	Agonist anti-CD40 antibody	APX005M	Phase II
		CFZ533	Phase II
		CP-870893	Phase I
		ADC-1013	Phase I
		Selicrelumab	Phase I
		Chi Lob 7/4	Phase I
**STING agonists**		MIW815	Phase I

CpG-ODN: CpG oligodeoxynucleotides; DC: dendritic cell; GM-CSF: granulocyte-macrophage colony-stimulating factor; IFN: interferon; IL-2: interleukin-2; MPLA: monophosphoryl lipid A; Poly-ICLC: polyinosinic-polycytidylic acid stabilized with polylysine and carboxymethylcellulose; STING: stimulator of interferon genes; TLR: Toll-like receptor.

**Table 4:** Examples of current immunomodulatory antibodies targeting T cells

Receptor	Ligand	Antibody	Stage of development
***Costimulation molecules***			
**4-1BB**	4-1BBL	Urelumab	Phase II
		Utomilumab	Phase I
		ADG106	Phase I
**OX40**	OX40L	MEDI6469	Phase II
		PF-04518600	Phase II
		GSK3174998	Phase I
		BMS 986178	Phase I
		MOXR0916	Phase I
		INBRX-106	Phase I
		BGB-A445	Phase I
**CD27**	CD70	Varlilumab	Phase II
**GITR**	GITRL	TRX518	Phase II
		BMS-986156	Phase II
		INCAGN01876	Phase II
		GWN323	Phase I
		MEDI1873	Phase I
		OMP-336B11	Phase I
		MK-4166	Phase I
**ICOS**	ICOSL	GSK3359609	Phase II
		Vopratelimab	Phase I/II
		KY1044	Phase I/II
**TNFRSF25**	TL1A		Preclinical
***Inhibitory molecules***			
**PD1**	PD-L1/PD-L2	Pembrolizumab	Approved
		Nivolumab	Approved
		Cemiplimab	Approved
		Sintilimab	Approved
		JS001	Approved
		Camrelizumab	Phase III
		BCD-100	Phase III
		Tislelizumab	Phase III
		Spartalizumab	Phase III
		Dostarlimab	Phase III
		REGN2810	Phase III
**CTLA4**	CD80/CD86	Ipilimumab	FDA approved
		Tremelimumab	Phase III
**LAG3**	MHC-II	Relatlimab	Phase II
		LAG525	Phase II
		REGN3767	Phase I
		TSR-033	Phase I
		Sym022	Phase I
**TIM3**	Phosphatidylserine	TSR-022	Phase II
		BGB-A425	Phase I/II
		MBG453	Phase I/II
		LY3321367	Phase I
		Sym023	Phase I

## Abbreviations

APCs: antigen-presenting cells
CAR-T cells: chimeric antigen receptor T cells
CpG-ODN: CpG oligodeoxynucleotides
CR: complete response
CTL: cytotoxic T lymphocyte
DC: dendritic cell
dLNs: draining lymph nodes
GM-CSF: granulocyte-macrophage colony-stimulating factor
GSH: glutathione
HNSCC: head and neck squamous cell carcinoma
HPV: human papillomavirus
IFA: incomplete Freund's adjuvant
IFN: interferon
IL-2: interleukin-2
MGMT: methylguanine methyltransferase
MHC: major histocompatibility complex
MHC-I molecules: MHC class I molecules
MHC-II molecules: MHC class II molecules
MPLA: monophosphoryl lipid A
MS: mass spectrometry
MSR: mesoporous silica micro-rod
ndLNs: non-draining lymph nodes
NGS: next-generation sequencing
NPs: nanoparticles
NSCLC: non-small cell lung cancer
OS: overall survival
PD: progressive disease
PEI: polyethyleneimine
PFS: progression-free survival
poly-ICLC: polyinosinic-polycytidylic acid stabilized with polylysine and carboxymethylcellulose
PR: partial response
PRR: pattern recognition receptor
RNA-Seq: RNA sequencing
SD: stable disease
sHDL: synthetic high-density lipoprotein
SLPs: synthetic long peptides
SNVs: single nucleotide variants
STING: stimulator of interferon genes
TAAs: tumor-associated antigens
TAP: transporter associated with antigen processing
T_c_ cells: cytotoxic T cells
TCR: T-cell receptor
T_h_ cells: T helper cells
TLR: Toll-like receptor
TMB: tumor mutation burden
TME: tumor microenvironment
TNBC: triple-negative breast carcinoma
WES: whole-exome sequencing
